# Effective Mobilization of Very Small Embryonic-Like Stem Cells and Hematopoietic Stem/Progenitor Cells but Not Endothelial Progenitor Cells by Follicle-Stimulating Hormone Therapy

**DOI:** 10.1155/2016/8530207

**Published:** 2015-11-09

**Authors:** Monika Zbucka-Kretowska, Andrzej Eljaszewicz, Danuta Lipinska, Kamil Grubczak, Malgorzata Rusak, Grzegorz Mrugacz, Milena Dabrowska, Mariusz Z. Ratajczak, Marcin Moniuszko

**Affiliations:** ^1^Department of Reproduction and Gynecological Endocrinology, Medical University of Bialystok, 15-276 Bialystok, Poland; ^2^Department of Regenerative Medicine and Immune Regulation, Medical University of Bialystok, 15-269 Bialystok, Poland; ^3^Department of Endocrinology, Diabetology and Internal Medicine, Medical University of Bialystok, 15-276 Bialystok, Poland; ^4^Department of Hematological Diagnostics, Medical University of Bialystok, 15-276 Bialystok, Poland; ^5^Center for Reproductive Medicine Bocian, 15-267 Bialystok, Poland; ^6^Stem Cell Institute at James Graham Brown Cancer Center, University of Louisville, Louisville, KY 40202, USA; ^7^Department of Regenerative Medicine, Medical University of Warsaw, 02-091 Warsaw, Poland; ^8^Department of Allergology and Internal Medicine, Medical University of Bialystok, 15-276 Bialystok, Poland

## Abstract

Recently, murine hematopoietic progenitor stem cells (HSCs) and very small embryonic-like stem cells (VSELs) were demonstrated to express receptors for sex hormones including follicle-stimulating hormone (FSH). This raised the question of whether FSH therapy at clinically applied doses can mobilize stem/progenitor cells in humans. Here we assessed frequencies of VSELs (referred to as Lin^−^CD235a^−^CD45^−^CD133^+^ cells), HSPCs (referred to as Lin^−^CD235a^−^CD45^+^CD133^+^ cells), and endothelial progenitor cells (EPCs, identified as CD34^+^CD144^+^, CD34^+^CD133^+^, and CD34^+^CD309^+^CD133^+^ cells) in fifteen female patients subjected to the FSH therapy. We demonstrated that FSH therapy resulted in statistically significant enhancement in peripheral blood (PB) number of both VSELs and HSPCs. In contrast, the pattern of responses of EPCs delineated by different cell phenotypes was not uniform and we did not observe any significant changes in EPC numbers following hormone therapy. Our data indicate that FSH therapy mobilizes VSELs and HSPCs into peripheral blood that on one hand supports their developmental origin from germ lineage, and on the other hand FSH can become a promising candidate tool for mobilizing HSCs and stem cells with VSEL phenotype in clinical settings.

## 1. Introduction

Maintenance of appropriate size and composition of both stem cell and progenitor cell pool is tightly regulated by continuous responding to surrounding and long-range orchestrating signals. Interestingly, sex hormones appeared lastly as important regulators of hematopoietic stem/progenitor cells (HSPCs) proliferation [1]. Recently, Nakada and colleagues revealed that hematopoietic stem cells (HSCs) expressed high levels of estrogen receptor and the administration of estradiol increased HSC cell division and self-renewal [2]. In support of this notion, murine HSPCs along with very small embryonic-like stem cells (VSELs) were also recently demonstrated to express receptors for pituitary-derived sex hormones, namely, follicle-stimulating hormone (FSH) and luteinizing hormone (LH) [3]. In concert with this finding, murine HSPCs and VSELs following either* in vitro* or* in vivo* FSH and LH stimulation presented with high proliferative response as evidenced by BrdU incorporation. In the light of above mentioned observations, it is tempting to hypothesize the existence of developmental link between HSCs and VSELs and primordial germ cells (PGCs) that are naturally responsive to sex hormones [4, 5].

To date, however, it remained unknown whether the fact that stem cells are susceptible to signaling mediated by sex hormones can be used for mobilization of these cells in clinical settings. Moreover, based on the currently available scarce data, it is difficult to speculate if therapies using sex hormones will affect only fate of primordial stem cells and HSCs or rather would exert their actions toward all progenitor cell populations. Therefore, in the current study, we wished to investigate the effects of FSH therapy at clinically applied doses on mobilization of HSCs and VSELs as well as populations of endothelial progenitor cells (EPCs). In this study, EPCs were chosen as an example of easily identifiable, highly differentiated, and relatively numerous progenitor cell populations that account for endothelial repair and thus largely contribute to maintenance of appropriate vasculature [6–8]. On the other hand, quantification of decreased numbers of EPCs was found to improve prognostication of cardiovascular diseases (CVD) [9–11]. Thus, the search for therapeutic approaches aimed at efficient mobilization of functional EPCs is continuously warranted.

Here we tested in human model the actions of widely accepted regimens of FSH treatment with regard to three stem/progenitor cell subsets at different developmental hierarchy and differentiation level, namely, VSELs, HSCs, and EPCs. Moreover, given the previous reports indicating the crucial role of stroma derived factor-1 (SDF-1) for mobilization of stem cells [12–14], we set out to analyze whether any actions of clinically applied gonadotropins could affect not only stem cells and progenitor cells but also mediators regulating their migratory pathways.

## 2. Material and Methods

### 2.1. Patients and FSH Stimulation

For the purpose of the study we recruited fifteen women aged 32.9 ± 3.9 years (range: 27–39 years) who were prepared for* in vitro* fertilization and underwent controlled FSH ovarian stimulation. FSH stimulation has been initiated on 3rd day of menstrual cycle and FSH dose was adjusted based on patient age, ovarian reserve, and previous response to FSH stimulation (if performed). Only two patients received stimulation based on combination of FSH and LH. EDTA-anticoagulated peripheral blood was collected twice: before FSH ovarian stimulation (or in five cases within first days of such stimulation) and at the end of FSH stimulation (days 7–11). Mean daily dose of FSH (either Gonal F, Merck Serono, or Puregon, Schering, or, in two patients, Menopur, Ferring) was 194.4 IU. Detailed characteristics of hormonal status of analyzed patients are presented in Table 1.

All patients' samples were collected upon the approval of Ethics Committee of the Medical University of Bialystok.

### 2.2. Extracellular Staining and Flow Cytometry

**Figure 1:**
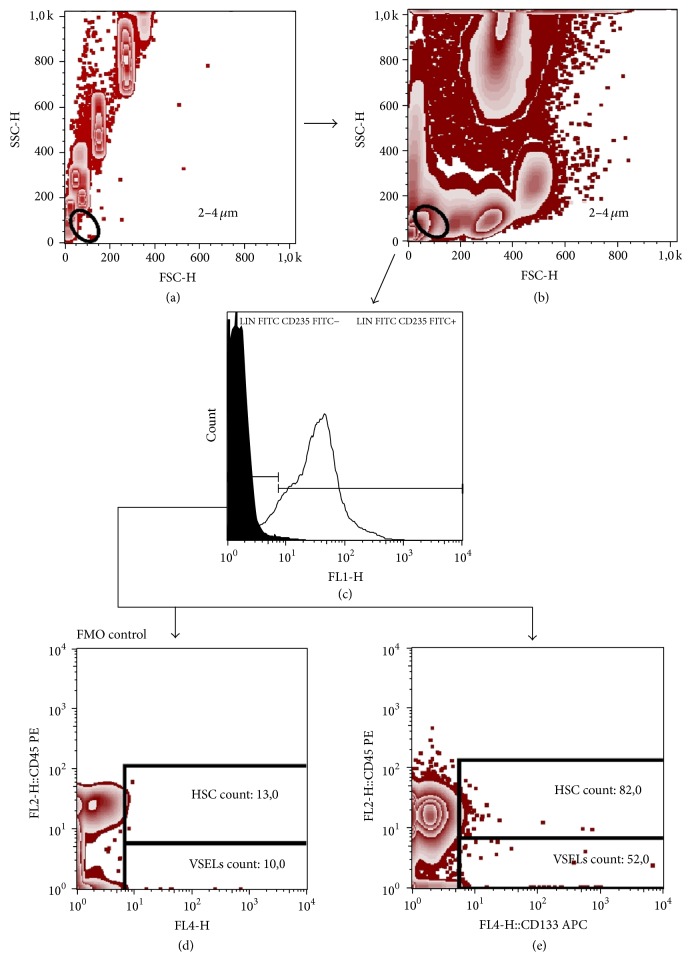
Representative FACS plots demonstrating gating strategy for HSC and VSELs. First, 2–4 *μ*m size events were gated based on a forward and side scatter (FSC/SSC) dot plot (a). Then the 2–4 *μ*m gate was visualized on sample data using a FSC/SSC dot plot (b). Next, 2–4 *μ*m events were displayed on histogram plot (black peak, FMO control; grey peak identifies positive staining) and Lin^−^CD235a^−^ events were gated (c). Finally, FMO control was used to set the HSC and VSELs gate and exclude the background noise (d). Next, HSCs were defined as Lin^−^CD235a^−^CD45^+^CD133^+^ cells (upper gate) and VSELs were referred to as Lin^−^CD235a^−^CD45^−^CD133^+^ cells (e).

**Figure 2:**
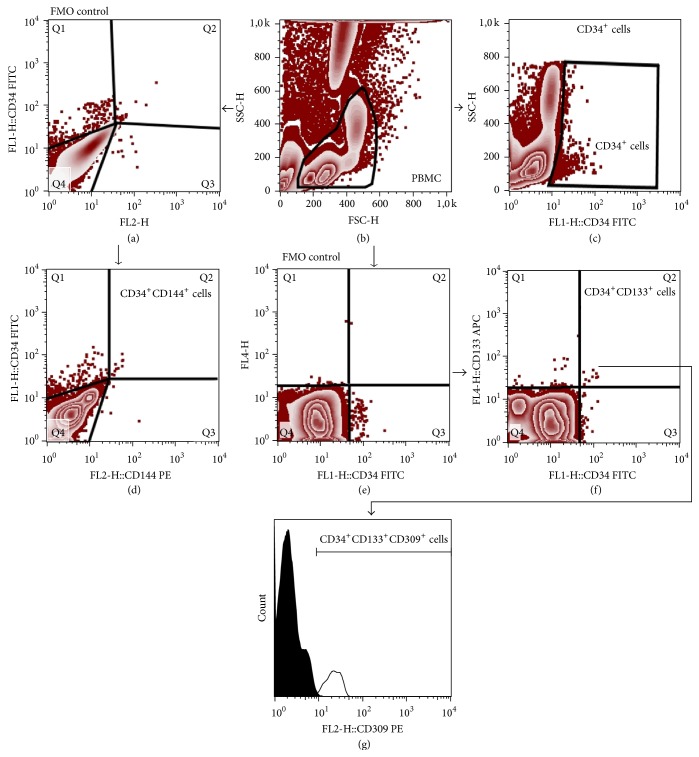
Representative FACS plots demonstrating the gating strategy for CD34^+^ cells and EPCs. PBMCs were gated based on forward and side scatter (FSC/SSC) plot (b). CD34^+^ cells were gated on CD34/SSC plot (c). CD34^+^CD144^+^ cells (upper right quadrant, Q2) were gated based on CD34/CD144 dot plot (d). FMO control was used to set the gates (a). In order to determine CD34^+^CD144^+^CD309^+^ cell numbers, the PBMC events were displayed on the basis of FMO control sample (e) and gates were set to exclude the random noise. Next, the gates were visualized on CD34/CD133 plot (f) and CD34^+^CD133^+^ cells were gated (upper right quadrant, Q2). Finally, CD34^+^CD133^+^CD309^+^ cells subsets were gated on histogram plot (g) (black peak, control; grey peak, positive staining).

170 *μ*L of fresh EDTA-anticoagulated whole blood was stained with the set of murine anti-human monoclonal antibodies described in detail in Table 2. Samples were incubated for 30 min at room temperature in the dark. Thereafter, 2 mL of FACS lysing solution (BD) was added, followed by 15 min incubation in the dark. Cells were washed twice with cold PBS (phosphate-buffered saline) and fixed with CellFix (BD Biosciences). Appropriate fluorescence-minus-one (FMO) controls were used for setting compensation and for assuring correct gating. The gating strategy for HSCs and VSELs is shown in Figure 1, while gating strategy for identifying CD34^+^ cells and EPCs is presented in Figure 2. Samples were acquired using FACSCalibur flow cytometer (BD Biosciences). Obtained data were analyzed using FlowJo version 7.6.5 software (Tree Star).

### 2.3. Enzyme-Linked Immunosorbent Assays

SDF-1 plasma levels were quantified by means of commercially available enzyme-linked immunosorbent assays (ELISA, DuoSet, R&D). Samples were directly assayed according to manufacturer's instructions. The protein levels in the specimens were calculated from a reference curve generated by using reference standards. The detection range of used ELISA set was between 7,81 and 500 pg/mL. The samples were analyzed with automated light absorbance reader (LEDETEC 96 system). Results were calculated by MicroWin 2000 software.

### 2.4. Statistics

Statistical analysis was carried out using GraphPad Prism 6 (GraphPad software). Wilcoxon test was used. The differences were considered statistically significant at *p* < 0.05. The results are presented as medians (interquartile range).

## 3. Results

**Figure 3:**
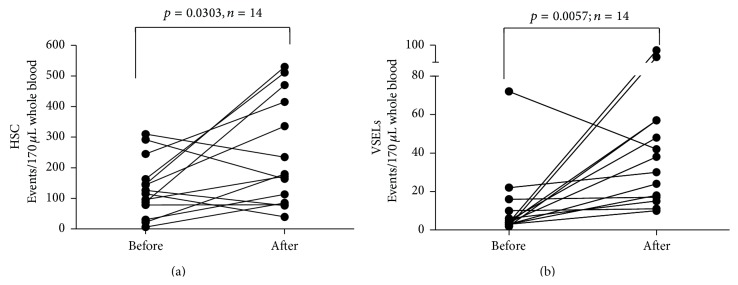
Summary of analyses of time-course changes of the numbers of HSCs (a) and VSELs (b) during FSH therapy.

First, we analyzed the effects of FSH administration on the frequencies of circulating HSCs (Figure 3(a)). We found that FSH treatment resulted in statistically significant increase in Lin^−^CD133^+^CD45^+^ HSC numbers ((from 125.5 (66–133.5) to 175.5 (83.5–428.8) cells per 170 *μ*L of whole blood), *p* = 0.0303). Similarly, FSH treatment led to significant enhancement of Lin^−^CD133^+^CD45^−^  VSEL levels (Figure 3(b)). Following FSH therapy, VSEL numbers increased from 4 cells (3–11,5) to 34 cells (16,5–57) per 170 *μ*L of whole blood (*p* = 0.0057).

**Figure 4:**
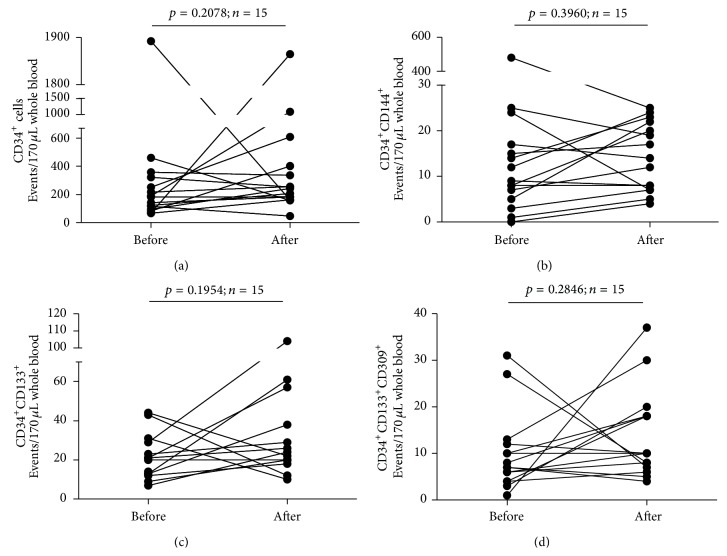
Summary of analyses of time-course changes of the numbers of CD34^+^ cells (a), CD34^+^CD144^+^cells (b), CD34^+^CD133^+^ cells (c), and CD34^+^CD133^+^CD309^+^ cells (d) in the course of FSH therapy.

Next, we set out to investigate the influence of FSH administration on the frequencies of entire population of CD34^+^ cells and the population of EPCs delineated by such phenotypes as CD34^+^CD144^+^, CD34^+^CD133^+^, and CD34^+^CD133^+^CD309^+^ cells. Notably, we did not observe any significant changes in the numbers of single CD34^+^ cells following FSH administration (Figure 4(a)). Similarly, we did not demonstrate any significant changes in numbers of EPCs identified as CD34^+^CD144^+^ cells (Figure 4(b)), CD34^+^CD133^+^ cells (Figure 4(c)), and CD34^+^CD133^+^CD309^+^ cells (Figure 4(d)).

**Figure 5:**
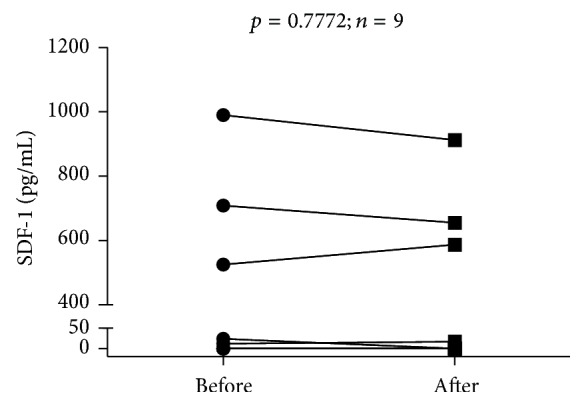
Time-course analysis of individual changes in serum levels of SDF-1 in the course of FSH therapy.

Finally, we wished to investigate whether FSH administration affected serum levels of SDF-1, one of crucial mobilizing factors for VSELs and HSCs (Figure 5). However, we did not find any significant change in SDF-1 concentrations upon completion of FSH administration (*p* > 0.05).

## 4. Discussion

Here we demonstrated that administration of pituitary-derived sex hormones at clinically applied doses allowed for efficient mobilization of frequencies of VSELs and HSCs but not endothelial progenitor cells. These findings build a platform for developing novel sex-hormone- based therapeutic strategies aimed at enhancement of either VSELs or HSCs numbers in conditions that would require efficient mobilization of these cells subsets.

Notably, it was shown that the links between gonadotropin hormones and stem cells may extend far beyond the effects exerted on VSELs and HSCs. Tadokoro and colleagues showed that FSH was one of crucial regulators of germinal stem cell (GSC) fate [15]. FSH regulated homeostatic control of glial cell line-derived neurotrophic factor (GDNF) which in turn accelerated proliferation of GSCs. These findings indicated that regulation of GCS population size is regulated by the GDNF/FSH pathway. It remains to be established whether effects of FSH treatment on VSELs and HSCs were related to actions mediated by GDNF. Recently, in concert with this notion, Tourkova and colleagues demonstrated that mesenchymal stem cells (MSC) expressing FSH-R responded with adhesion and proliferation following addition of FSH [16]. Moreover, short-term treatment by FSH at doses comparable to those observed in menopause augmented MSC proliferation by affecting signaling associated with Erk1/2 phosphorylation.

It remains elusive whether mobilizing effects of FSH/LH therapy on VSELs and HSCs in female patients were related to their direct effects on bone marrow-derived stem cells or rather on mobilization of stem cells localized in ovaries. On the other hand, both possibilities do not have to be mutually exclusive. Previously, it was demonstrated that stem cells found in ovaries have the phenotype of VSELs and they can give rise to more differentiated ovarian GCS [17, 18]. This was confirmed by later studies by Parte and colleagues who demonstrated that VSELs and GSCs were present in ovary surface epithelium (OSE) [19]. In this study, FSH treatment resulted in prominent proliferation of stem cell-harboring OSE and release of functional stem cells from ovaries. Moreover, Patel and colleagues found that FSH treatment resulted in increased clonal expansion of ovary-associated stem cells (both VSELs and ovarian GCSs), while FSH receptors were expressed only on ovarian stem cells but not on ovarian epithelial cells [20]. Similarly, Sriraman and colleagues showed that PMSG (FSH analog) increased VSELs numbers in chemoablated female mice [21]. Nevertheless, the question of how effects of gonadotropin hormones on stem cell population size are related to ovaries would require enrollment of male patients subjected to therapy with FSH and/or LH, the condition that due to ethical reasons certainly could not be achieved in the settings of the current study.

Notably, in our study, we did not demonstrate any significant effects of gonadotropin therapy on the levels of EPCs. In concert with this notion, in a representative group of patients with inflammatory bowel disease, Garolla and colleagues did not find relationships between levels of FSH or LH and numbers of EPCs [22]. In some contrast, however, reduced numbers of EPCs were found in hypogonadotropic hypogonadal male patients presenting with low levels of FSH and LH [23]. Interestingly, receptor for FSH is expressed by the endothelium of blood vessels in the majority of metastatic tumors [24]. Therefore, despite lack of significant effects of gonadotropin therapy on numbers of circulating EPCs analyzed in the current study, the subject of mutual relationships among EPCs, endothelial cells, and pituitary-derived sex hormones certainly deserves further investigation.

Our findings also bring about an interesting perspective for understanding relationships between stem and progenitor cells and elevated levels of FSH and LH that are detected at elderly age. In the light of our data, it is tempting to hypothesize that enhanced FSH and LH levels in elderly individuals could represent the mechanism of enhancing otherwise diminished hematopoiesis and decreased stem cell numbers. Similarly, this observation was previously documented in mouse model, wherein the numbers of VSELs were shown to be highest in young subjects and decreased with age [25].

Given these data, FSH and LH therapy could become an attractive and easily available tool enabling mobilization of stem cells in regenerative medicine. However, given the broad spectrum of the effects exerted by sex hormones, the safety of such approach would need to be examined in further clinical studies performed in different groups of patients including males. One has to keep in mind that FSH and LH were found to be involved in the growth of certain tumors. As an example, Ji and colleagues found significantly higher levels of mRNA encoding receptor for FSH (FSHR) in invasive ovarian tumors compared to low malignant tumors and normal OSE [26]. Similarly, it was reported that overexpression of FSHR in OSE cells led to an increase in expression of proteins involved in ovarian cancer development such as EGFR, c-myc, and HER2/neu. Thus any therapeutic strategies aimed at enhancement of the size of stem/progenitor cell pool by the use of gonadotropin-based therapies would need to be carefully investigated in terms of clinical safety.

Altogether, our data support recent findings on the role of FSH in the biology of VSELs and HSCs that were reported by our group in mouse model [4]. Thus, these data support a concept of a developmental link between germ line, VSELs, and hematopoiesis [4, 5]. Finally, we demonstrated here for the first time that mobilization of stem cells with VSELs phenotype and HSPCs can be achieved by the use of widely available therapeutic regimens based on pituitary-derived sex hormones.

## Figures and Tables

**Table 1:** The clinical and hormonal characteristics of female patients (*n* = 15) subjected to FSH stimulation.

Studied parameter	Mean	SD	Minimum	Maximum
Age (years)	32.9	3.9	27	39
Duration of stimulation (days)	8.8	1.1	8	11
Mean daily dose of FSH (IU)	194.4	43.8	120	262.5
Number of mature follicles after FSH stimulation	10.9	3.4	5	16
Estradiol at 7th day (pg/mL)	1153	405	540	1684
Progesterone at 7th day (ng/mL)	0.5	0.15	0.25	0.67
LH at 7th day (mIU/mL)	1.1	0.67	0.31	2.33
Estradiol at the last day (pg/mL)	2314	1367	1367	3294
Progesterone at the last day (ng/mL)	0.8	0.33	0.32	1.52
LH at the last day (mIU/mL)	1.7	0.85	0.72	2.97

**Table 2:** Detailed characteristics of monoclonal antibodies used in the study.

Name	Clone	Isotype	Format	Additional information	Manufacturer
Mouse anti-human anti-CD34	8G12	IgG1	FITC	This antibody binds to 105–120 kDa single-chain transmembrane glycoprotein, IVD	Becton Dickinson
Anti-human lineage cocktail 2 (lin 2)			FITC		Becton Dickinson
Mouse anti-human anti-CD3	SK7	IgG1		This antibody binds to epsilon chain of the CD3 antigen, IVD	
Mouse anti-human anti-CD19	SJ25C1	IgG1		This antibody recognizes a 90 kDa antigen, IVD	
Mouse anti-human anti-CD20	L27	IgG1		This antibody binds to phosphoprotein with a molecular weight of 35 or 37 kilodaltons (kDa), depending on the degree of phosphorylation, IVD	
Mouse anti-human anti-CD14	M_*φ*_P9	IgG2b		This antibody reacts with a 53–55 kDa glycosylphosphatidylinositol- (GPI-) anchored and single chain glycoprotein, IVD	
Mouse anti-human anti-CD56	NCAM16.2	IgG2b		This antibody recognizes a heavily glycosylated 140 kDa isoform of NCAM, a member of the immunoglobulin (Ig) superfamily, IVD	
Mouse anti-human anti-CD235a	GA-R2	IgG2b	FITC	This antibody binds to glycophorin A, a sialoglycoprotein present on human red blood cells (RBC) and erythroid precursor cells	Becton Dickinson
Mouse anti-human anti-CD45	HI30	IgG1	PE	This antibody binds to 190, 190, 205, and 220 kDa protein isoforms, RUO	Becton Dickinson
Mouse anti-human anti-CD144	55-7H1	IgG1	PE	This antibody reacts with calcium-independent epitope on cadherin 5, RUO	Becton Dickinson
Mouse anti-human anti-CD309	89106	IgG1	PE	This antibody reacts with CD309 (vascular endothelial growth factor receptor-2 (VEGFR-2))	Becton Dickinson
Mouse anti-human anti-CD133	AC133	IgG1	APC	This antibody reacts with epitope 1 of CD133, RUO	Miltenyi Biotec

IVD: this clone is used for *in vitro* diagnostics; RUO: suitable for research use only.

## References

[1] Gancz D., Gilboa L. (2013). Hormonal control of stem cell systems. *Annual Review of Cell and Developmental Biology*.

[2] Nakada D., Oguro H., Levi B. P. (2014). Oestrogen increases haematopoietic stem-cell self-renewal in females and during pregnancy. *Nature*.

[3] Mierzejewska K., Borkowska S., Suszynska E. (2015). Hematopoietic stem/progenitor cells express several functional sex hormone receptors—novel evidence for a potential developmental link between hematopoiesis and primordial germ cells. *Stem Cells and Development*.

[4] Ratajczak M. Z., Marycz K., Poniewierska-Baran A., Fiedorowicz K., Zbucka-Kretowska M., Moniuszko M. (2014). Very small embryonic-like stem cells as a novel developmental concept and the hierarchy of the stem cell compartment. *Advances in Medical Sciences*.

[5] Scaldaferri M. L., Klinger F. G., Farini D. (2015). Hematopoietic activity in putative mouse primordial germ cell populations. *Mechanisms of Development*.

[6] Rusak M., Radzikowska U., Glowinska-Olszewska B. (2015). Endothelial progenitor cell levels in juvenile idiopathic arthritis patients; effects of anti-inflammatory therapies. *Pediatric Rheumatology*.

[7] Khakoo A. Y., Finkel T. (2005). Endothelial progenitor cells. *Annual Review of Medicine*.

[8] Goerke S. M., Obermeyer J., Plaha J., Stark G. B., Finkenzeller G. (2015). Endothelial progenitor cells from peripheral blood support bone regeneration by provoking an angiogenic response. *Microvascular Research*.

[9] Głowińska-Olszewska B., Moniuszko M., Hryniewicz A. (2013). Relationship between circulating endothelial progenitor cells and endothelial dysfunction in children with type 1 diabetes: a novel paradigm of early atherosclerosis in high-risk young patients. *European Journal of Endocrinology*.

[10] Lavoie J. R., Stewart D. J. (2012). Genetically modified endothelial progenitor cells in the therapy of cardiovascular disease and pulmonary hypertension. *Current Vascular Pharmacology*.

[11] Sen S., McDonald S. P., Coates P. T. H., Bonder C. S. (2011). Endothelial progenitor cells: novel biomarker and promising cell therapy for cardiovascular disease. *Clinical Science*.

[12] Ratajczak M. Z., Kim C. H., Abdel-Latif A. (2012). A novel perspective on stem cell homing and mobilization: review on bioactive lipids as potent chemoattractants and cationic peptides as underappreciated modulators of responsiveness to SDF-1 gradients. *Leukemia*.

[13] Kucia M., Reca R., Miekus K. (2005). Trafficking of normal stem cells and metastasis of cancer stem cells involve similar mechanisms: pivotal role of the SDF-1-CXCR4 axis. *Stem Cells*.

[14] Kucia M., Ratajczak J., Reca R., Janowska-Wieczorek A., Ratajczak M. Z. (2004). Tissue-specific muscle, neural and liver stem/progenitor cells reside in the bone marrow, respond to an SDF-1 gradient and are mobilized into peripheral blood during stress and tissue injury. *Blood Cells, Molecules, and Diseases*.

[15] Tadokoro Y., Yomogida K., Ohta H., Tohda A., Nishimune Y. (2002). Homeostatic regulation of germinal stem cell proliferation by the GDNF/FSH pathway. *Mechanisms of Development*.

[16] Tourkova I. L., Witt M. R., Li L. (2015). Follicle stimulating hormone receptor in mesenchymal stem cells integrates effects of glycoprotein reproductive hormones. *Annals of the New York Academy of Sciences*.

[17] Parte S., Bhartiya D., Telang J. (2011). Detection, characterization, and spontaneous differentiation in vitro of very small embryonic-like putative stem cells in adult mammalian ovary. *Stem Cells and Development*.

[18] Virant-Klun I., Zech N., Rozman P. (2008). Putative stem cells with an embryonic character isolated from the ovarian surface epithelium of women with no naturally present follicles and oocytes. *Differentiation*.

[19] Parte S., Bhartiya D., Manjramkar D. D., Chauhan A., Joshi A. (2013). Stimulation of ovarian stem cells by follicle stimulating hormone and basic fibroblast growth factor during cortical tissue culture. *Journal of Ovarian Research*.

[20] Patel H., Bhartiya D., Parte S., Gunjal P., Yedurkar S., Bhatt M. (2013). Follicle stimulating hormone modulates ovarian stem cells through alternately spliced receptor variant FSH-R3. *Journal of Ovarian Research*.

[21] Sriraman K., Bhartiya D., Anand S., Bhutda S. (2015). Mouse ovarian very small embryonic-like stem cells resist chemotherapy and retain ability to initiate oocyte-specific differentiation. *Reproductive Sciences*.

[22] Garolla A., D’Incà R., Checchin D. (2009). Reduced endothelial progenitor cell number and function in inflammatory bowel disease: a possible link to the pathogenesis. *American Journal of Gastroenterology*.

[23] Foresta C., de Toni L., Selice R., Garolla A., di Mambro A. (2010). Increased osteocalcin-positive endothelial progenitor cells in hypogonadal male patients. *Journal of Endocrinological Investigation*.

[24] Siraj A., Desestret V., Antoine M. (2013). Expression of follicle-stimulating hormone receptor by the vascular endothelium in tumor metastases. *BMC Cancer*.

[25] Kucia M., Reca R., Campbell F. R. (2006). A population of very small embryonic-like (VSEL) CXCR4^+^SSEA-1^+^Oct-4^+^ stem cells identified in adult bone marrow. *Leukemia*.

[26] Ji Q., Liu P. I., Chen P. K., Aoyama C. (2004). Follicle stimulating hormone-induced growth promotion and gene expression profiles on ovarian surface epithelial cells. *International Journal of Cancer*.

